# Real-World Functional and Anatomical Outcomes of Intravitreal Faricimab in Suboptimal Responders With Neovascular Age-Related Macular Degeneration: A 24-Month Clinical Audit

**DOI:** 10.7759/cureus.111161

**Published:** 2026-06-19

**Authors:** Dhanya Sukumaran, Delicia Jayakumar, Mohamed Naved Abdul Karim, Ruby Amankwah, Prema Maharajan, Munir Mubashar, Ambreen Sarmad, Priyanka Sharma, Sushma Dhar-Munshi

**Affiliations:** 1 Department of Ophthalmology, King's Mill Hospital, Sherwood Forest Hospitals NHS Foundation Trust, Sutton-in-Ashfield, GBR

**Keywords:** angiopoietin-2, anti-vegf, faricimab, intravitreal injection, namd, neovascular age-related macular degeneration, optical coherence tomography, real-world outcomes, treatment burden

## Abstract

Introduction: Neovascular age-related macular degeneration (nAMD) is a chronic sight-threatening disease requiring repeated intravitreal antivascular endothelial growth factor (anti-VEGF) injections. Although established anti-VEGF agents have transformed the prognosis of nAMD, a proportion of patients demonstrate persistent exudation, limited durability, or inability to extend beyond short treatment intervals. Faricimab is a bispecific intravitreal antibody targeting both vascular endothelial growth factor A and angiopoietin-2, designed to improve vascular stability, reduce exudation, and increase treatment durability. This clinical audit evaluates 24-month real-world outcomes following switching to faricimab in suboptimal responders with nAMD in an NHS medical retina service.

Methods: A retrospective clinical audit was conducted of 274 eyes with nAMD switched to faricimab at Sherwood Forest Hospitals NHS Foundation Trust between January 2023 and January 2025. All eyes had previously received at least one anti-VEGF agent and were switched because of persistent disease activity, inadequate anatomical response, or high treatment burden. Data were extracted from the Medisight electronic patient record and cross-checked against pharmacy records. Primary outcomes were best corrected visual acuity (BCVA) in Early Treatment Diabetic Retinopathy Study letters and central macular thickness (CMT) on optical coherence tomography. Secondary outcomes included injection interval extension, number of injections, and safety events.

Results: Baseline mean BCVA was 58.1 letters. Mean BCVA improved to 59.7 letters at less than six months, representing a gain of +1.6 letters. At 12 months, mean BCVA was 56.9 letters, a change of -1.2 letters from baseline. At 24 months, mean BCVA was 54.4 letters, a change of -3.7 letters from baseline. Anatomical response was more consistent, with mean CMT reductions of -29.1 µm at less than six months, -34.7 µm at 12 months, and -45.7 µm at 24 months. The mean injection interval increased from 7.88 weeks before switching to 10.67 weeks after switching, representing an extension of +2.79 weeks. No cases of intraocular inflammation, retinal vasculitis, or endophthalmitis were recorded.

Conclusion: In this real-world NHS audit, faricimab achieved meaningful anatomical improvement and reduced treatment burden in treatment-experienced nAMD eyes switched because of suboptimal response or limited durability on previous anti-VEGF therapy. Visual acuity improved modestly during early follow-up but declined slightly over 24 months, despite progressive anatomical drying. This functional-anatomical dissociation likely reflects the chronicity of disease and irreversible macular damage in a switch population. Faricimab appears to be a safe and effective second-line option for reducing exudation and extending treatment intervals, although expectations regarding long-term visual gain should be realistic.

## Introduction

Neovascular age-related macular degeneration (nAMD) is a leading cause of irreversible central visual loss in older adults. The introduction of intravitreal antivascular endothelial growth factor (anti-VEGF) therapy has substantially improved outcomes, converting nAMD from a rapidly blinding disease into a chronic condition requiring long-term monitoring and repeated treatment. Ranibizumab, aflibercept, and bevacizumab have all been widely used in clinical practice, and treat-and-extend regimens are now commonly employed to balance disease control against the burden of frequent injections.

Despite these advances, several challenges remain. A significant subgroup of patients has persistent intraretinal fluid, subretinal fluid, or pigment epithelial detachment despite regular treatment. Others remain anatomically controlled only at short intervals, often four to eight weeks, creating a high cumulative burden for patients, carers, imaging services, injection clinics, and pharmacy departments. In NHS medical retina services, the pressure generated by repeated intravitreal therapy is substantial. Therefore, treatments that improve durability without compromising vision are clinically valuable.

Faricimab is a bispecific antibody designed for intraocular use. Unlike conventional anti-VEGF monotherapy, it targets both vascular endothelial growth factor A (VEGF-A) and angiopoietin-2. VEGF-A contributes to vascular permeability and neovascularization, while angiopoietin-2 is implicated in vascular destabilization, inflammation, and increased sensitivity to VEGF-mediated leakage. By inhibiting both pathways, faricimab aims to improve vascular stability and reduce exudation. The pivotal TENAYA and LUCERNE trials demonstrated that faricimab was noninferior to aflibercept in treatment-naïve nAMD, with a substantial proportion of patients maintained on extended dosing intervals up to every 16 weeks [1,2]. National Institute for Health and Care Excellence subsequently recommended faricimab as an option for treating wet AMD in eligible adults in the United Kingdom [3].

However, pivotal clinical trial populations differ from real-world switch cohorts. Patients in routine NHS practice often have longstanding disease, multiple prior injections, macular atrophy, fibrosis, or incomplete anatomical response. These features can limit visual recovery even when retinal fluid improves. Real-world evidence is, therefore, essential to determine whether the anatomical and durability benefits observed in clinical trials translate into routine care. Recent real-world studies, including UK datasets such as FARWIDE-nAMD and Moorfields switch cohorts, suggest that faricimab can maintain vision, improve anatomical outcomes, and extend injection intervals in treatment-experienced eyes [4,5].

This audit evaluates the 24-month functional, anatomical, durability, and safety outcomes of faricimab in patients with nAMD switched from prior anti-VEGF therapy at Sherwood Forest Hospitals NHS Foundation Trust. The aim was to determine whether switching to faricimab achieved clinically meaningful disease control and reduced treatment burden in a real-world NHS setting.

## Materials and methods

Study design and setting

This was a retrospective clinical audit conducted within the Medical Retina Department at Sherwood Forest Hospitals NHS Foundation Trust. The audit evaluated consecutive eyes with nAMD that were switched to intravitreal faricimab between January 2023 and January 2025. As this work was conducted as a service evaluation and clinical audit using routinely collected, anonymized clinical data, formal research ethics approval was not required under local audit governance processes. The audit was conducted in accordance with institutional information governance standards.

Study population

The audit included 274 eyes diagnosed with nAMD that had received at least one faricimab injection after previous treatment with another intravitreal anti-VEGF agent. Eyes were switched at the discretion of the treating clinician. Common reasons for switching included persistent intraretinal or subretinal fluid, recurrent fluid despite regular therapy, inability to extend treatment interval, high injection burden, or a clinical impression of suboptimal response to previous therapy.

Eyes were included if they had a confirmed diagnosis of nAMD, a record of previous anti-VEGF therapy, baseline best corrected visual acuity (BCVA) and optical coherence tomography (OCT) data at or near the time of switch, and at least one subsequent faricimab treatment episode. Eyes were excluded from relevant time-point analyses if follow-up data were unavailable at that time point. Therefore, cohort numbers varied over time: 274 eyes contributed to the less-than-six-month analysis, 243 eyes to the 12-month analysis, and 127 eyes to the 24-month analysis.

Data source and variables

Data were extracted retrospectively from the Medisight electronic patient record system. A live Excel spreadsheet (Microsoft Corporation, Redmond, WA) was populated using clinical records, injection records, OCT measurements, and treatment interval data. Pharmacy records were cross-referenced to confirm faricimab administration and improve data completeness.

The following variables were collected: baseline BCVA, BCVA at follow-up time points, baseline and follow-up central macular thickness (CMT), the number of faricimab injections, pre-switch treatment interval, post-switch treatment interval, and recorded adverse events. Safety outcomes included intraocular inflammation, retinal vasculitis, infectious endophthalmitis, and any other documented severe ocular adverse event.

Outcome measures

The primary functional outcome was change in BCVA, measured in Early Treatment Diabetic Retinopathy Study (ETDRS) letters. The primary anatomical outcome was change in CMT on OCT, measured in micrometers. Follow-up outcomes were grouped into three clinically relevant intervals: less than six, 12, and 24 months.

Secondary outcomes were treatment durability and safety. Durability was assessed using the mean treatment interval before switching and the mean treatment interval after switching to faricimab. Treatment burden was further described using mean injection numbers across follow-up windows: less than six, 7-12, and 12-24 months.

Statistical analysis

Descriptive statistics were used for this audit. Mean BCVA, mean CMT change, mean injection interval, and mean injection number were calculated for each time point. Because this was a retrospective service audit rather than a hypothesis-driven clinical trial, no formal comparative statistical testing was performed. Outcomes were interpreted clinically in the context of treatment-experienced switch populations.

## Results

Cohort and follow-up

A total of 274 eyes with nAMD were included at baseline. All eyes had been treated previously with at least one anti-VEGF agent before switching to faricimab. Follow-up data were available for 274 eyes at less than six months, 243 eyes at 12 months, and 127 eyes at 24 months. The reduction in numbers over time reflected variable follow-up duration, transfer of care, discontinuation, death, or incomplete data capture, which are common features of real-world retrospective datasets.

Functional outcomes

Baseline mean BCVA was 58.1 ETDRS letters. At less than six months, mean BCVA improved to 59.7 letters, representing a gain of +1.6 letters from baseline. This suggests a modest early functional response following switch, possibly reflecting improved exudative control in a subset of eyes.

**Figure 1:**
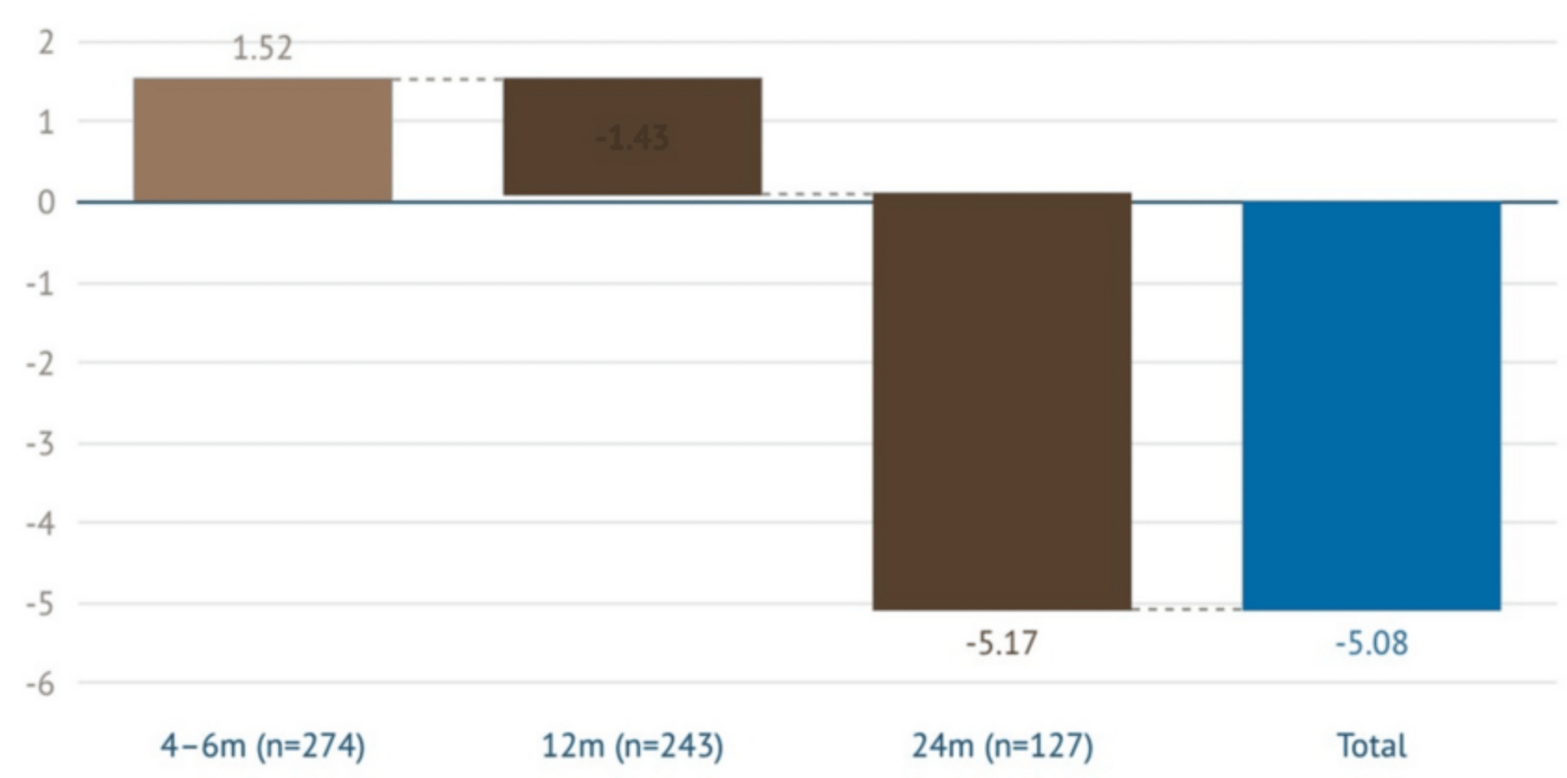
Mean vision change from baseline

At 12 months, mean BCVA was 56.9 letters. This represented a reduction of -1.2 letters compared with baseline. At 24 months, mean BCVA was 54.4 letters, representing a reduction of -3.7 letters from baseline (Figure 1). The visual trajectory therefore showed early stabilization or mild improvement followed by gradual decline over longer follow-up.

The magnitude of visual decline at 24 months was modest but clinically relevant. In a chronic switch cohort, this decline may reflect underlying disease progression rather than treatment failure alone. Potential contributors include photoreceptor disruption, subretinal fibrosis, macular atrophy, cataract progression, and cumulative structural damage from longstanding nAMD.

Anatomical outcomes

**Figure 2:**
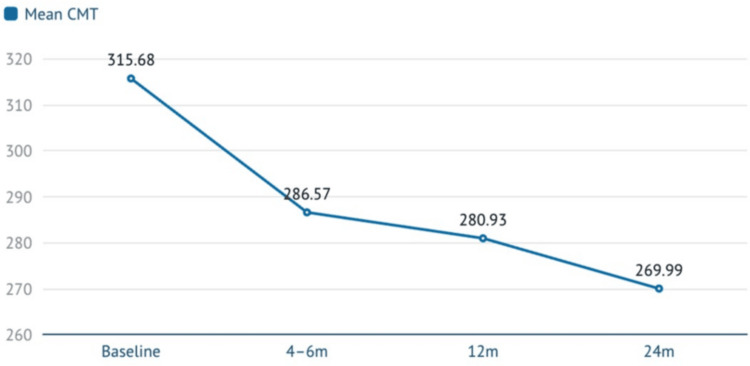
Mean CMT CMT: central macular thickness

In contrast to the functional trend, anatomical outcomes showed consistent improvement. Mean CMT reduced by -29.1 µm at less than six months, -34.7 µm at 12 months, and -45.7 µm at 24 months (Figure 2). This progressive reduction suggests that faricimab achieved sustained anatomical drying in many eyes that had previously demonstrated persistent or recurrent exudation on other anti-VEGF agents.

The increasing magnitude of CMT reduction over time is notable. It indicates that some eyes may continue to derive anatomical benefit beyond the initial loading or early switch phase. In clinical practice, this supports the role of faricimab as a useful option for patients with persistent intraretinal or subretinal fluid despite established therapy.

Treatment burden and durability

The mean treatment interval before switching was 7.88 weeks. Following switch to faricimab, the mean interval increased to 10.67 weeks. This represents a mean extension of +2.79 weeks (Table 1).

**Table 1:** Prior-switch interval vs. post-switch interval

Treatment interval	Values
Mean prior switch interval (weeks)	7.88
Mean post switch interval (weeks)	10.67
Mean difference (weeks)	2.79
Median difference (weeks)	2.00

This interval extension is clinically important. In a high-volume NHS macular service, even a two- to three-week increase in average interval can reduce annual injection numbers, OCT appointments, nursing workload, pharmacy preparation, and patient travel burden. The observed extension suggests that faricimab may help address capacity pressures while maintaining anatomical disease control.

Mean faricimab injection numbers were 4.65 injections during the less-than-six-month period, 2.84 injections between seven and 12 months, and 4.79 injections between 12 and 24 months. These figures are consistent with a transition from early intensive treatment to longer maintenance intervals in selected patients.

Safety outcomes

No cases of intraocular inflammation, retinal vasculitis, or endophthalmitis were recorded among the 274 eyes included in this audit. This represents a reassuring safety signal in routine clinical practice. However, because rare adverse events require large sample sizes to estimate accurately, the absence of recorded events in this cohort should not be interpreted as proof of zero risk.

## Discussion

This 24-month clinical audit demonstrates that faricimab can deliver sustained anatomical improvement and reduce treatment burden in treatment-experienced nAMD eyes switched because of suboptimal response or limited durability on prior anti-VEGF therapy. The key finding is a clear functional-anatomical dissociation: CMT improved progressively over time, while BCVA showed only early modest gain followed by gradual decline.

The early BCVA gain of +1.6 letters at less than six months suggests that some patients benefited functionally after switching. This may reflect reduction in active exudation, improved retinal architecture, or better control of intraretinal fluid. However, by 12 months, the mean BCVA had fallen slightly below baseline, and by 24 months, the mean loss was -3.7 letters. In the context of chronic nAMD, this degree of decline is not unexpected. Visual acuity in longstanding nAMD is determined not only by the presence or absence of fluid but also by irreversible structural damage, including outer retinal atrophy, retinal pigment epithelial disturbance, subretinal hyperreflective material, and fibrosis.

The anatomical response was more robust. Mean CMT reductions increased from -29.1 µm at early follow-up to -45.7 µm at 24 months. This suggests that faricimab was effective at reducing exudation even in eyes previously considered difficult to control. Similar patterns have been reported in real-world switch studies, where anatomical improvement is often more pronounced than visual improvement [4-11]. This distinction is important when counseling patients. In treatment-naïve eyes, macula drying may be associated with meaningful visual gain. In chronic switch eyes, the more realistic goal may be stabilization, prevention of further exudative damage, and reduction in injection burden.

The observed extension of the interval from 7.88 to 10.67 weeks is highly relevant to NHS service delivery. A mean extension of +2.79 weeks may translate into fewer injections per year and fewer hospital attendances. For elderly patients, many of whom depend on relatives, carers, hospital transport, or community support, fewer appointments may improve quality of life and adherence. For services, interval extension may release clinical capacity for new referrals, urgent reviews, and patients requiring frequent therapy.

The safety findings were reassuring, with no recorded cases of intraocular inflammation or endophthalmitis. Published faricimab studies generally report low rates of ocular inflammation and serious ocular adverse events [1,2,4,8,10,11]. Nevertheless, ongoing vigilance is essential. Rare adverse events may not appear in a cohort of this size, and real-world pharmacovigilance remains important as treatment exposure increases.

Comparisons with the pivotal TENAYA and LUCERNE trials must be made with caution. Those trials enrolled treatment-naïve nAMD patients under protocolized conditions, whereas this audit included treatment-experienced eyes switched because of suboptimal response or high treatment burden. The better visual outcomes in clinical trials are, therefore, not directly comparable. More relevant comparators are real-world switch studies. The Moorfields one-year study reported maintenance of visual acuity, improved anatomical outcomes, and extended treatment intervals in treatment-intensive nAMD eyes switched to faricimab [5]. FARWIDE-nAMD, a large UK real-world study, similarly supports the ability of faricimab to extend intervals and prevent vision loss in previously treated eyes [4].

Several limitations should be acknowledged. First, the retrospective design limits control over data completeness, imaging intervals, and treatment decisions. Second, the cohort experienced attrition over time, with 274 eyes at baseline but 127 eyes contributing to 24-month outcomes. This may introduce selection bias, as patients with better or worse outcomes may be more likely to remain under follow-up, discontinue, or be switched again. Third, the audit did not capture several potentially important confounders, including the duration of nAMD before the switch, the number of previous injections, lens status, cataract surgery, baseline fibrosis, macular atrophy, lesion subtype, fluid compartment, or fellow-eye status. Fourth, no formal statistical testing was performed; therefore, the findings should be interpreted descriptively. Finally, no formal correlation analysis between changes in central macular thickness and best corrected visual acuity (BCVA) was performed, limiting assessment of the structure-function relationship at an individual eye level.

Despite these limitations, the audit has strengths. It reflects routine NHS practice, includes a relatively large treatment-experienced cohort, and provides 24-month outcome data, which remain limited in published real-world switch literature. The findings are clinically relevant because they describe the type of patient commonly encountered in medical retina clinics: elderly, previously treated, chronically active, and often unable to extend successfully on conventional anti-VEGF therapy.

The results support faricimab as a valuable second-line agent in nAMD. The main benefit appears to be anatomical drying and interval extension rather than sustained visual gain, consistent with long-term anti-VEGF outcomes in large trials such as Comparison of AMD Treatments Trials showing plateauing visual outcomes despite ongoing treatment burden [12]. Real-world evidence from large cohorts further demonstrates that injection intensity strongly influences visual outcomes in nAMD [13]. Faricimab’s role as a recently approved dual-pathway agent is well established in regulatory and clinical literature [14]. This does not diminish its clinical value. In chronic nAMD, maintaining functional vision while reducing treatment burden is a meaningful outcome for patients and services. Real-world switch data also support improved durability and anatomical response in treatment-experienced nAMD eyes switched to faricimab [15].

## Conclusions

Faricimab was effective and well tolerated as a second-line switch therapy for nAMD in this real-world NHS clinical audit. It produced progressive anatomical improvement, with mean CMT reductions reaching -45.7 µm at 24 months, and extended mean treatment intervals by +2.79 weeks. Visual acuity improved modestly during early follow-up but declined by -3.7 letters at 24 months, despite continued anatomical drying.

These findings suggest that, in treatment-experienced suboptimal responders, faricimab should be viewed primarily as a therapy to improve anatomical control, stabilize disease activity, and reduce treatment burden. Patients should be counseled that visual improvement may be limited by chronic macular damage, but fewer injections and a drier retina are realistic and clinically meaningful goals. Further prospective studies with detailed OCT biomarkers, lens status, fibrosis grading, and atrophy assessment would help identify which switch patients are most likely to derive functional benefit.
